# Supplementary material for: Vaginal Dysbiosis in Infertility: A Comparative Analysis Between Women with Primary and Secondary Infertility
Source: Microorganisms. 2025 Jan 17;13(1):188. doi: 10.3390/microorganisms13010188 (PMC11767898; doi:10.3390/microorganisms13010188)
Supplement: Supplementary file 1 [file microorganisms-13-00188-s001.zip › microorganisms-3342559-supplementary.pdf]

Table S1. Primer and probe sequences used to detect target microorganisms

Organism	Sequence target	Primer sequence
Lactobacillus jensenii	Strain SNUV360 Chromosome (GenBank ID: CP018809.1, CP046310.1)	F:5´-TTCGGTGACTGGAGTGATATTG<br>R: 5´-AGGTAGAGGTCCTTGTACTTACT<br>P: CATGATCAACCACTTATCAAAGCACTCTGA-FAM
Lactobacillus crispatus	16S ribosomal RNA	F: 5´-AACTAACAGATTTACTTCGGTAATGA<br>R: 5´-AGCTGATCATGCGATCTGC<br>P: CCCATAGTCTGGGATACCCTT-FAM
Lactobacillus gasseri	16S ribosomal RNA	F: 5´-CGAGCTTGCCTAGATGAATTTG<br>R: 5´-TCCAGGTGTTATCCCAGTCTC<br>P: ACTAGATACAAGCGAGCGGC-FAM
Lactobacillus iners	Gen *hem*H = Ferroquelatasa (GenBank ID: CP049230.1, CP045664.1, CP049228.1, CP0492231.1)	F: 5´-TGTGGATTAATGCCTTGGTTAAAG<br>R: 5´-GCAATAGTTTCAGAACAATCGGTAA<br>P: TCCAAGCGGCTAAAGACGGTATGA-FAM
Mobiluncus mulieris	Gen *met*G (GenBank ID: UGPX01000007.1, JACHMA010000001.1)	F: 5´-CTGGCGACCCAATGTGATTA<br>R: 5´-CGGCATCAAACCACACATAAAG<br>P: AGGAAATTAAGCCTCGCGCCATGA-FAM
Gardnerella vaginalis	Heat shock protein 60 (*hsp*60) gene sequence (NCBI GenBank Access: AF240579.3)	F: 5´-AATCTCTGGTGCACGAAGGC-3´<br>R: 5´-ACATCCTTAGCAGATGCGAGA-3´<br>P: AGCAACCCGATCGCTCTTCGTCGCGGA-FAM
Fannyhessea vaginae	16S ribosomal RNA sequence of strain VCE255 (NCBI GenBank Access number: MH628052.1)	F: 5´-CCTTACCAGGGCTTGACATTTA-3´<br>R: 5´-CGGGACTTAACCCAACATCTC-3´<br>P: AAGGAGCCTAAACAGGTGGTGCAT-FAM

F: forward primer. R: reverse primer, P: probe

Table S2. Logistic Regression: Primary Infertility

	Std.	Error	z	Pr(>|z|)	
CST	1.29354	0.65264	1.982	**0.04748**	*
AGE	0.08786	0.04289	2.048	**0.04052**	*
Sexually transmitted bacteria	-0.61071	0.48442	-1.261	0.20742	
VPH	0.99563	0.58085	1.714	0.08651	.
Cryptococcus neoformans	15.29862	2399.545	0.006	0.99491	
Epstein–Barr Virus	0.50509	0.79928	0.632	0.52743	
Cytomegalovirus	0.54472	0.76178	0.715	0.47457	
Escherichia coli K1	-16.41695	1228.73479	-0.013	0.98934	
Streptococcus agalactiae	0.4971	0.6732	0.738	0.46026	
Haemophilus influenzae	-1.00198	0.86241	-1.162	0.2453	
Herpes virus	0.27916	0.97095	0.288	0.77372	
Gardnerella vaginalis	1.241	0.5529	2.244	**0.0248**	*
Fannyhessea vaginae	1.31294	0.73453	1.787	0.07386	.
Mobiluncus mulieris	-0.43635	0.92881	-0.47	0.63851	
Lactobacillus jensenii	2.84047	1.4487	1.961	**0.04991**	*
Lactobacillus crisspatus	3.56789	2.09019	1.707	0.08783	.
Lactobacillus iners	1.62596	1.04152	1.561	0.11849	
Lactobacillus gasseri	2.74608	1.42852	1.922	0.05457	.

Signif. codes: 0 ‘***’ 0.001 ‘**’ 0.01 ‘*’ 0.05 ‘.’ 0.1 ‘ ’ 1

Table S3. Logistic Regression: Secondary Infertility

	Std.	Error	z	Pr(>|z|)	
CST	-0.89645	0.565	-1.587	0.1126	
AGE	-0.03223	0.03691	-0.873	0.3826	
Sexually transmitted bacteria	0.40238	0.49725	0.809	0.4184	
VPH	-0.13086	0.53272	-0.246	0.806	
Cryptococcus neoformans	13.18265	2399.545	0.005	0.99256	
Epstein–Barr Virus	-1.25898	0.81838	-1.538	0.124	
Cytomegalovirus	-0.21582	0.79904	-0.27	0.7871	
Escherichia coli K1	16.29754	1684.08283	0.01	0.9923	
Streptococcus agalactiae	-0.6139	0.6651	-0.923	0.356	
Haemophilus influenzae	1.74428	0.98879	1.764	0.0777	.
Herpes virus	1.31164	1.07117	1.224	0.2208	
Gardnerella vaginalis	-1.52077	0.60776	-2.502	**0.0123**	*
Fannyhessea vaginae	0.35623	0.64859	0.549	0.5828	
Mobiluncus mulieris	0.45124	0.83055	0.543	0.5869	
Lactobacillus jensenii	-0.81415	1.24104	-0.0656	0.5118	
Lactobacillus crisspatus	-2.04916	1.84364	-1.111	0.2664	
Lactobacillus iners	0.14207	0.93238	0.152	0.8789	
Lactobacillus gasseri	-0.77929	1.18818	-0.656	0.5119	

Signif. codes: 0 ‘***’ 0.001 ‘**’ 0.01 ‘*’ 0.05 ‘.’ 0.1 ‘ ’ 1

Table S4. PERMANOVA Bray–Curtis

	Df	Sum of Squares	R^2	F Pr(>F)
CST	4	0.53173 - 0.18675	7.5206	0.001
Residual	131	2.31552 - 0.81325		
Total	135	2.84725 - 1.00000		

Number of permutations: 999

Signif. codes: 0 ‘***’ 0.001 ‘**’ 0.01 ‘*’ 0.05 ‘.’ 0.1 ‘ ’ 1

Table S5. Sexually transmitted bacteria versus all variables

	Std.	Error	z	Pr(>|z|)	
HPV	1.08974	0.52612	2.071	0.0383	*

Signif. codes: 0 ‘***’ 0.001 ‘**’ 0.01 ‘*’ 0.05 ‘.’ 0.1 ‘ ’ 1

Table S6. *Gardnerella vaginalis versu*s all variable

	Std.	Error	z	Pr(>|z|)	
Sexually transmitted bacteria	1.51108	0.69584	2.172	0.02989	*
Epstein–Barr Virus	-2.19835	1.14756	-1.916	0.05541	.
Haemophilus influenzae	1.72685	0.92505	1.867	0.06194	.
Fannyhessea vaginae	2.2346	0.71474	3.126	0.00177	**

Signif. codes: 0 ‘***’ 0.001 ‘**’ 0.01 ‘*’ 0.05 ‘.’ 0.1 ‘ ’ 1
